# Clinical-Scale Production of CAR-T Cells for the Treatment of Melanoma Patients by mRNA Transfection of a CSPG4-Specific CAR under Full GMP Compliance

**DOI:** 10.3390/cancers11081198

**Published:** 2019-08-16

**Authors:** Manuel Wiesinger, Johannes März, Mirko Kummer, Gerold Schuler, Jan Dörrie, Beatrice Schuler-Thurner, Niels Schaft

**Affiliations:** Department of Dermatology, Universtitätsklinikum Erlangen, Friedrich-Alexander-Universität Erlangen-Nürnberg, Hartmannstraße 14, 91052 Erlangen, Germany

**Keywords:** CAR-T cell, melanoma, CSPG4, clinical scale production, full GMP compliance, clinical study, consistency runs

## Abstract

Chimeric antigen receptor (CAR)-T cells already showed impressive clinical regressions in leukemia and lymphoma. However, the development of CAR-T cells against solid tumors lags behind. Here we present the clinical-scale production of CAR-T cells for the treatment of melanoma under full GMP compliance. In this approach a CAR, specific for chondroitin sulfate proteoglycan 4 (CSPG4) is intentionally transiently expressed by mRNA electroporation for safety reasons. The clinical-scale protocol was optimized for: (i) expansion of T cells, (ii) electroporation efficiency, (iii) viability, (iv) cryopreservation, and (v) potency. Four consistency runs resulted in CAR-T cells in clinically sufficient numbers, i.e., 2.4 × 10^9^ CAR-expressing T cells, starting from 1.77x10^8^ PBMCs, with an average expansion of 13.6x, an electroporation efficiency of 88.0% CAR-positive cells, a survival of 74.1% after electroporation, and a viability of 84% after cryopreservation. Purity was 98.7% CD3^+^ cells, with 78.1% CD3^+^/CD8^+^ T cells and with minor contaminations of 1.2% NK cells and 0.6% B cells. The resulting CAR-T cells were tested for cytolytic activity after cryopreservation and showed antigen-specific and very efficient lysis of tumor cells. Although our work is descriptive rather than investigative in nature, we expect that providing this clinically applicable protocol to generate sufficient numbers of mRNA-transfected CAR-T cells will help in moving the field of adoptive cell therapy of cancer forward.

## 1. Introduction

Autologous T cells, reprogrammed to target malignant cells via the expression of a chimeric antigen receptor (CAR-T cells) represent a promising tool in the adoptive cellular therapy of cancer. Impressive clinical regressions of leukemias or lymphomas have been achieved using CD19-specific CAR-T cells in several clinical trials. This culminated in the approval by the FDA and EMA of Kymriah^®^ (Tisagenlecleucel), a one-time treatment for B-cell acute lymphoblastic leukemia (ALL) that has shown an 83% remission rate after three months in clinical trials with patients that do not respond to standard treatments, and Yescarta^®^ (Axicabtagen-Ciloleucel), which induced remissions in 72% of the patients with aggressive B-cell non-Hodgkin lymphoma [1].

However, most clinical trials focus on the elimination of these so-called liquid tumors; the development of CAR-T cells against solid tumors lags behind (reviewed in [2,3,4,5]). This is due to the lack of real tumor-specific antigens that can be targeted by CAR-T cells, causing potential on-target/off-tumor toxicity due to the accidental killing of non-malignant bystander cells co-expressing the target antigen [6]. The expression of the target antigen on healthy tissue always bears the risk of severe side effects due to tissue toxicity. This is probably the reason that very few CAR-T cells against different antigens expressed on melanoma (e.g., VEGFR2, CD70, GD2, c-Met) were tested in clinical trials (NCT03060356, NCT01218867, NCT02107963, NCT02830724).

Chondroitin sulfate proteoglycan 4 (CSPG4), also known as melanoma-associated- chondroitin-sulfate-proteoglycan (MCSP), high molecular weight-melanoma-associated antigen (HMW-MAA), or neuron-glial antigen 2 (NG2) is a 450 kDa large, heavily glycosylated proteoglycan [7,8]. CSPG4 is expressed on almost all melanoma cells [9], but also on uveal melanoma [10,11], and on other tumors like sarcomas, astrocytomas, gliomas, neuroblastomas [12,13,14,15], leukemias [16,17,18,19,20], and triple negative breast cancer [21]. In many of those malignancies, CSPG4-expression is associated with poor prognosis and aggressive growth [22]. On non-pathologic tissue, CSPG4 is expressed on precursors of hair-follicle and epidermis cells, as well as on endothelial cells and on activated pericytes, however, not on mature vasculature [23,24]. Moreover, CSPG4 is expressed on chondrocytes of the articular cartilage [25], on smooth muscle cells [26], on brain pericytes [27], and on cells of the neuromuscular synapse of human postnatal skeleton muscles [28]. The antigen is also expressed on fetal melanocytes, but not on healthy melanocytes of adults [29]. The expression of CSPG4 on healthy tissues is, however, clearly weaker than on tumor cells [8,30,31]. Nevertheless, CSPG4 is a prime tumor target antigen [30], since it plays a role in the metastasizing of melanoma [32], and is expressed on activated pericytes during angiogenesis in tumors and hypoxia [33,34,35], the latter making targeting of tumor vasculature possible. CSPG4-specific monoclonal antibodies [36], radio-immunoconjugates [37], or immunotoxins [38,39,40] were already applied in animal models and melanoma patients, with partially promising results [41]. Other strategies to specifically eliminate CSPG4-positive targets include fusion proteins linking a CSPG4 binding domain to soluble TRAIL (TNF-related apoptosis-inducing ligand) agonists to initiate cell death upon CSPG4 binding through the extrinsic apoptosis pathway [42].

T cells, virally transduced with a CSPG4-specific CAR, exerted potent cytotoxicity in response to various CSPG4-expressing tumors, such as melanoma, breast cancer, mesothelioma, glioblastoma and osteosarcoma [43,44,45,46,47,48,49] in animal models or in vitro. Additionally, intracranial application of CSPG4-CAR T cells in a murine model of glioblastoma imposed efficient tumor control [50].

To circumvent concerns about potential on-target/off-tumor toxicities, we have previously demonstrated that transient transfection of T cells with CSPG4-CARs using mRNA electroporation might be an effective and safe tool in cancer immunotherapy [51,52,53]. Using RNA-transfected CAR-T cells offers the advantage that the receptor expression is temporally restricted, rendering potential off-target and on-target/off-tumor toxicity transient as well. For safety reasons, an initial use of repetitive injections of RNA-transfected CSPG4-CAR-T cells may be beneficial to probe for toxicity. In the case of no serious side-effects, a switch to permanently transfected CSPG4-CAR-T cells may be conceivable.

No CSPG4-specific CAR-T cells have been used in humans so far. Therefore, it was our aim in this study to establish the clinical-scale production of CAR-T cells for the treatment of melanoma patients by mRNA transfection of a CSPG4-specific CAR under full GMP compliance, in direct preparation of a clinical trial using these cells. To show the robustness of our production process, which is a prerequisite to obtain a manufacturing license, we performed four consistency runs using the optimized protocol for CAR-T cell production. We were able to repeatedly produce a sufficient number of highly pure CSPG4-CAR-transfected T cells with a very high transfection efficiency, a high CAR expression, and a high potency to kill melanoma target cells. This protocol will be used in the very near future for the production of CAR-T cells for a clinical phase I trial in melanoma patients.

## 2. Results

### 2.1. Sufficient Numbers of CAR-Transfected T Cells Are Generated from PBMCs Originating from Leukaphereses

**Figure 1:**
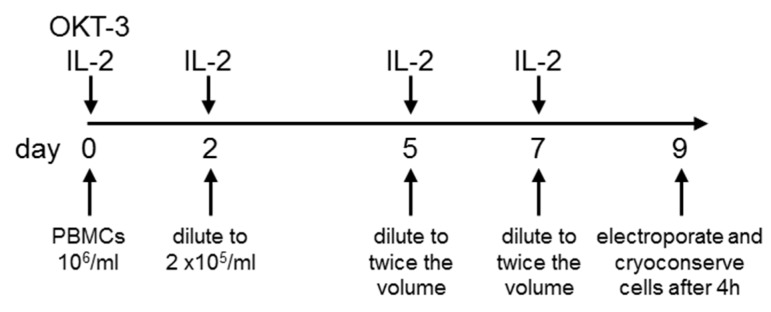
Schematic representation of the expansion and electroporation procedure. PBMCs were isolated from leukaphereses by density centrifugation using Lymphoprep^TM^ and were expanded for nine days in X-vivo 15 medium in bags with a starting concentration of 1 × 10^6^ cells/mL. OKT-3 (0.1 µg/mL) was added at day zero. IL-2 was added at day zero (1000 IU/mL), day two (1000 IU/mL), day five (500 IU/mL), and day seven (250 IU/mL). Cells were diluted at day two to 2 × 10^5^ cells/mL, and at day five and seven, the volume of the X-vivo 15 medium is doubled. At day nine, the cells were harvested and 3.24 × 10^9^ cells were electroporated with mRNA encoding the CSPG4-specific CAR. Four hours after that, the CAR-transfected T cells were cryoconserved in batches of 45 × 10^6^ cells. For technical details of this protocol, please see Section 4.

We previously published a protocol to generate CAR-T cells by mRNA electroporation [54]. Although this protocol was intended for GMP-translation, the actual upscaling and practical performance under original GMP conditions required a further adaption and optimization. Thus, the main question in this study was: Is it possible to generate sufficient numbers of highly functional CAR-RNA T cells under full GMP compliance? Therefore, we optimized the expansion/electroporation protocol for several parameters: (1) expansion rate, (2) electroporation efficiency, (3) viability, (4) ability to cryopreserve, and (5) potency. The most optimal protocol considering all these factors consisted of expansion of T cells from PBMCs isolated from leukaphereses for nine days in X-vivo 15 medium in bags (Figure 1). At day zero, OKT-3 and IL-2 are added (Figure 1). At day two, cells are diluted to 2 × 10^5^ cells/mL and IL-2 is added (Figure 1). At day five and seven, the cells are diluted to twice the volume and IL-2 is added (Figure 1). Finally, at day nine, the cells are harvested and electroporated with mRNA encoding the CSPG4-specific CAR (Figure 1). After four hours, the CAR-transfected T cells are cryoconserved in batches suitable for application to the patient. For technical details of this protocol, please see Section 4 and Supplementary Figures S2–S4.

Four consistency runs were performed using this protocol to test the robustness of the procedure. Leukaphereses for consistency run one, two, and three were performed with individual donors. For consistency run four we performed an additional leukapheresis of the same donor as for consistency run one. Data of these four consistency runs are summarized in Table 1. On average 4.67 × 10^9^ cells were harvested at day nine of the expansion, starting from an average of 2.37x10^8^ PBMCs on day zero (Table 1). For technical reasons (see Section 3), the maximum number of cells that could be electroporated with mRNA encoding the CSPG4-specific CAR was 3.24 × 10^9^ cells. The electroporation resulted in 2.4 × 10^9^ CAR-transfected T cells, which corresponds to a survival rate after electroporation of 74.1% (Table 1). The survival rate after electroporation and cryoconservation was 58.4% (Table 1). Taken all the data together, the yield factor, i.e., the number of CAR-transfected T cells at day nine divided by the number of PBMCs at day zero, was on average 13.6 (Table 1).

This means that 2.4 × 10^9^ CAR-expressing T cells were generated starting from 1.77 × 10^8^ PBMCs. This is a sufficient number of CAR-T cells to treat melanoma patients.

### 2.2. T Cells Are Preferentially Expanded over 9 Days

**Figure 2:**
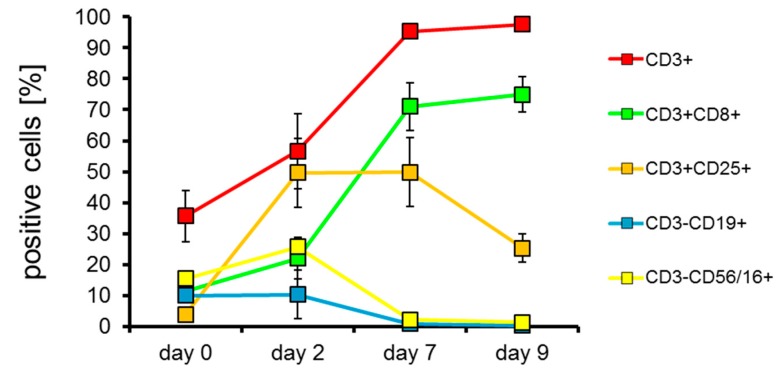
Phenotype of the cells during the expansion over nine days. PBMCs were expanded as described in Figure 1 in four consistency runs. At day zero, two, seven, and nine the phenotype of the cells was determined by measuring CD3, CD8, CD25, CD19, and CD56/CD16 expression using corresponding antibodies. The mean percentages of positive cells of the four consistency runs +/− SEM are shown.

To get an impression on how T cells were expanded specifically in our protocol using OKT-3 and IL-2 as stimulating agents, we determined the phenotype on day zero, two, seven, and nine of all four consistency runs. As can be seen in Figure 2, the proportion of CD3^+^ and CD3^+^/CD8^+^ cells increases over the nine days of expansion. Furthermore, CD25 expression on CD3^+^ cells increases on the expanded cells initially, probably induced by the addition of IL-2, but levels out from day two to seven, and even decreases from day seven to nine (Figure 2). Most importantly, the portion of contaminating B cells (CD3^−^CD19^+^) and NK cells (CD3^-^CD56/16^+^) decreased during the expansion over nine days (Figure 2). Looking at the cell populations at the end of the expansion period at day nine in more detail, we saw that 97.7% were T cells (CD3^+^), 75.0% were CD8-positive T cells (CD3^+^CD8^+^), 25.3% of the T cells were expressing CD25 (CD3^+^CD25^+^), 0.3% were contaminating B cells (CD3^−^CD19^+^), and 1.3% were contaminating NK cells (CD3^−^CD56/16^+^) (Figure 2). Moreover, CD14+ cells (i.e., monocytes) had completely vanished, and on average 12.5% CD3^+^CD56/16^+^ (i.e., NKT cells) were present at day nine of expansion (data not shown).

**Figure 3:**
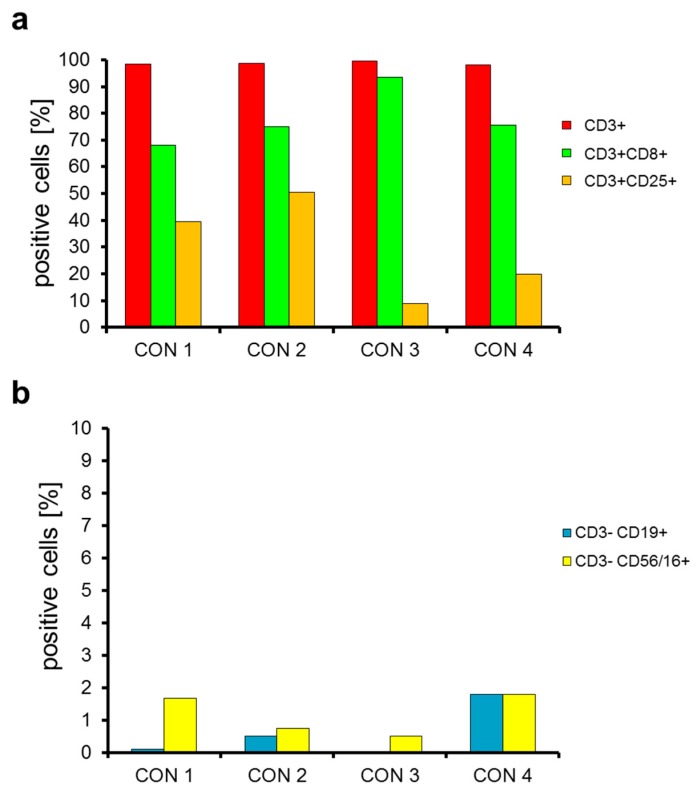
Phenotype of the chimeric antigen receptor (CAR)-transfected cells after expansion over nine days, electroporation and cryoconservation. PBMCs were expanded, electroporated, and cryoconserved as described in Figure 1 in four consistency runs. After several days, the cells were thawed, and 4 h after thawing the phenotype of the cells was determined by measuring CD3 (**a,b**), CD8 (**a**), CD25 (**a**), CD19 (**b**), and CD56/CD16 (**b**) expression using corresponding antibodies. The percentages of positive cells of all four consistency runs are shown.

These expanded cells were then electroporated and cryoconserved in vials containing small portions of 45 × 10^6^ CAR-transfected T cells for dose escalation reasons (see discussion). After several days, these cells were thawed. The CAR-transfected T cells had an average viability of 84% (Supplementary Figure S1) and a yield of 58.4% (Table 1) after electroporation and cryoconservation. We also determined the phenotype of the CAR-transfected cells after cryoconservation (Figure 3). We found that 98.7% were T cells (CD3^+^; Figure 3a), 78.1% were CD8-positive T cells (CD3^+^CD8^+^; Figure 3a), 29.7% of the T cells were expressing CD25 (CD3^+^CD25^+^; Figure 3a), 0.6% were contaminating B cells (CD3^−^CD19^+^; Figure 3b), and 1.2% were contaminating NK cells (CD3^−^CD56/16^+^; Figure 3b). In addition, we determined the CD4-positive fraction in these expanded, electroporated and cryoconserved CAR-T cells (Supplementary Figure S5). On average 15.9% of the cells were CD3^+^CD4^+^. Furthermore, 5.0% of the living cells were CD3^+^CD4^+^ and CD25^+^ (Supplementary Figure S5). Due to the preceding anti-CD3 stimulation, most of these cells probably represent activated CD4^+^ T cells, which also express CD25 [55], and not regulatory T cells.

Taken together, we can conclude that the expanded and then CAR-transfected cells were mostly T cells and that there is a low contamination of other cells in the product.

### 2.3. The CSPG4-Specific CAR Is Expressed Very Efficiently on T Cells Electroporated with CAR-Encoding mRNA

**Figure 4:**
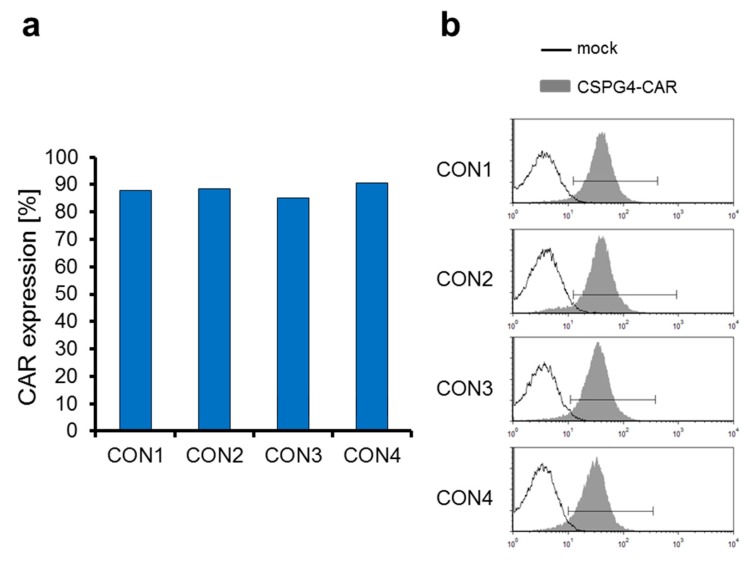
Transfection efficiency of mRNA encoding the chondroitin sulfate proteoglycan 4 (CSPG4)-specific CAR. PBMCs were expanded, electroporated, and cryoconserved as described in Figure 1 in four consistency runs. As a control mock-electroporated cells were generated. After several days, the cells were thawed, and 4 h after thawing the CAR expression on the cell surface of the T cells was determined using a PE-labeled goat anti-human IgG antibody, binding the Fc-part of the CAR (**a**,**b**). The percentages of positive cells of all four consistency runs are shown (**a**). Furthermore, histograms of the CAR expression are shown (**b**).

One of the aims of the optimization of the procedure for clinical-scale production of CAR-T cells was to obtain a high transfection efficiency. After the expansion for nine days, 3.24 × 10^9^ of the resulting cells were electroporated with in vitro transcribed mRNA encoding the CSPG4-specific CAR. Four hours after electroporation, the cells were frozen in small batches of 45 × 10^6^ cells per vial. After several days these cells were thawed again, and four hours after thawing the expression of the CSPG4-specific CAR was determined by staining of the extracellular IgG1 CH2CH3 (Fc-spacer) CAR-domain with goat-F(ab’)2 anti-human IgG antibody. The average transfection efficiency in the four consistency runs was 88.0% (Figure 4a).

In addition, we investigated the heterogeneity of the CAR expression on mRNA-transfected T cells. The histograms of the CAR stainings indicate that the CAR expression was actually quite homogenous (Figure 4b), which is in line with our previous observations with electroporation of several different mRNAs in several different cell types.

In summary, we conclude that electroporation of CSPG4-CAR-encoding mRNA into T cells results in a high transfection efficiency and a homogenous expression of the CAR on the cell surface.

### 2.4. CSPG4-CAR-Transfected T Cells Show a Very High Potency to Lyse Melanoma Target Cells

**Figure 5:**
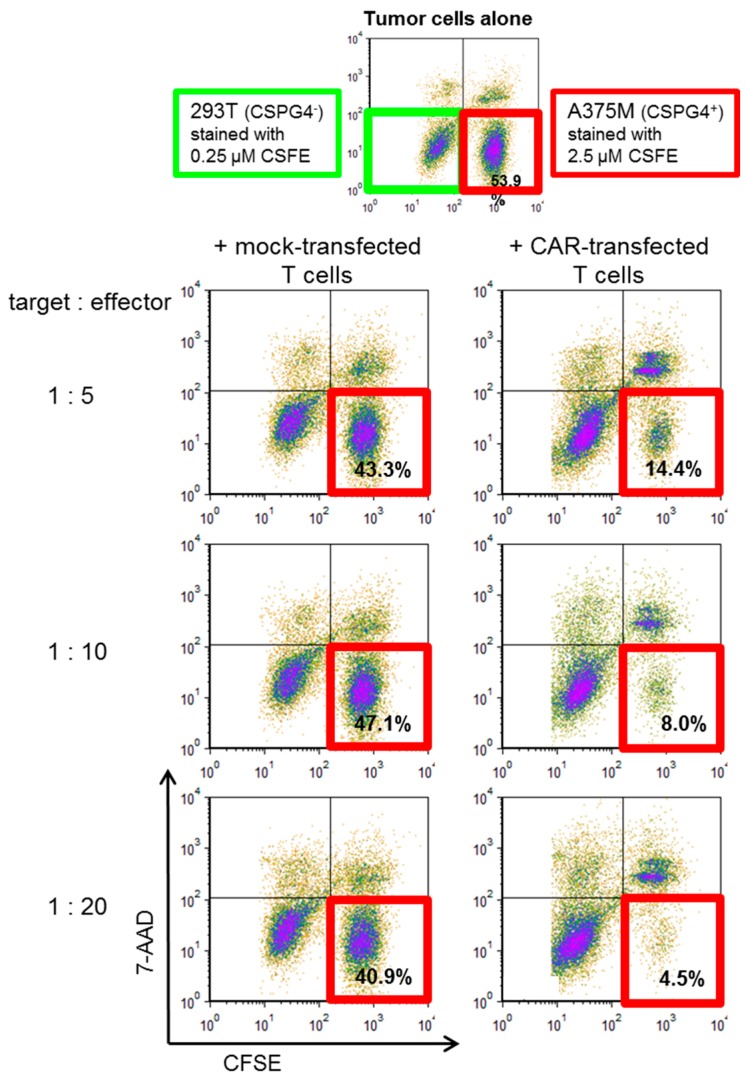
Lysis of target cells by CSPG4-CAR-transfected T cells. PBMCs were expanded, electroporated, and cryoconserved as described in Figure 1 in four consistency runs. As a control mock-electroporated cells were generated. After several days, the cells were thawed and used in a cytotoxicity assay. As target cells the CSPG4-negative 293T cell line and the CSPG4-positive A375M melanoma cell line were used. 293T cells were labeled with 0.25 µM CFSE and A375M cells were labeled with 2.5 µM CFSE. These cells were mixed at a 1:1 ratio. Target cells and effector cells were co-incubated for 20 h at indicated target: effector ratios. Then cells were harvested and stained with 7-AAD. CFSE and 7-AAD staining was determined by flow cytometry. All surviving cells, i.e., 293T CFSE^low^, 7-AAD negative cells and A375M CFSE^high^, 7-AAD negative cells, were set to 100%. The proportion of A375M cells in all surviving cells are indicated in the dot blots. Shown is representative data of one consistency run. For gating strategy see Supplementary Figure S6.

The most important function of CAR-T cells is the lysis of tumor target cells. To investigate the cytolytic capacity of the CSPG4-CAR-transfected T cells generated in the four consistency runs, we established a cytotoxicity assay based on differential CFSE labeling of target cells to be used as a potency assay. We labeled the CSPG4-negative target cell line 293T with 0.25 µM CFSE and the CSPG4-positive melanoma cell line A375M with 2.5 µM CFSE. These labeled cells were mixed at a 1 to 1 ratio (Figure 5; upper panel). T cells generated with our expansion protocol were either mock electroporated or electroporated with mRNA encoding the CSPG4-specific CAR. These effector cells were added to the target cells at indicated ratios (Figure 5; representative data with T cells of one consistency run are shown). After 20 h of co-incubation, all cells were harvested, stained with 7-AAD, and the 7-AAD and CFSE staining was analyzed by flow cytometry (Figure 5). Mock-transfected T cells hardly induced lysis of the A375M melanoma cells at all target to effector ratios (Figure 5). Of all surviving target cells, >40% were A375M melanoma cells (i.e., CFSE^high^; Figure 5). In contrast, CSPG4-CAR-transfected T cells had a very high potency to lyse the melanoma cells at all target to effector ratios. Of all surviving target cells, only 4.5% were A375M melanoma cells at a target to effector ratio of 1:20 (Figure 5). A dose dependency was observed, i.e., the percentage of living A375M cells decreased by increasing the effector cells (Figure 5).

**Figure 6:**
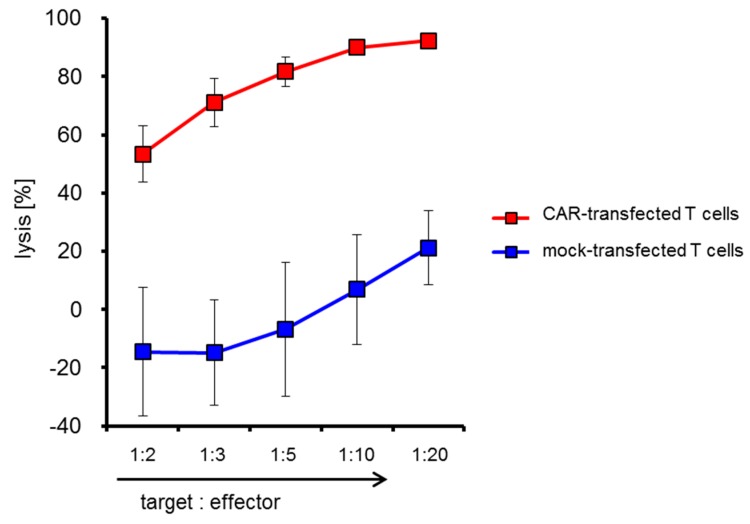
Lysis of A375M melanoma cells by mock- and CAR-transfected T cells. PBMCs were expanded, electroporated, and cryoconserved as described in Figure 1 in four consistency runs. As a control mock-electroporated cells were generated. After several days, the cells were thawed and used in a cytotoxicity assay. As target cells the CSPG4-negative 293T cell line and the CSPG4-positive A375M melanoma cell line were used. 293T cells were labeled with 0.25 µM CFSE and A375M cells were labeled with 2.5 µM CFSE. These cells were mixed at a 1:1 ratio. Target cells and effector cells were co-incubated for 20 h at indicated target: effector ratios. Then, cells were harvested and stained with 7-AAD. CFSE and 7-AAD staining was determined by flow cytometry. The percentage of lysis (i.e., cytotoxicity induced by T cells and background apoptosis) of A375M cells was calculated as follows, with A being: $A\;=\frac{\%\;living\;A375M\;cells}{\%\;living\;293T\;cells}$
and percentage of lysis being:$\frac{A\;\%\;\left[target\;alone\right]-A\;\%\;\left[target\;+\;effector\right]}{A\;\%\;\left[target\;alone\right]}\times \;100\%$

In addition, we calculated the percentage of lysis of A375M melanoma cells by either mock-transfected or CSPG4-CAR-transfected T cells of all consistency runs and plotted the mean +/− SEM in Figure 6. Already at a target to effector ratio of 1:2 the CAR-transfected T cells were able to lyse the melanoma cells (Figure 6). A background lysis by mock-transfected T cells is also observed in higher effector to target ratios (Figure 6). The percentage of lysis increases when adding more effector cells, culminating in a specific lysis (i.e., lysis induced by CAR-transfected T cells—background lysis induced by mock-transfected T cells) of on average 71.1% at a target to effector ratio of 1:20 (Figure 6).

**Figure 7:**
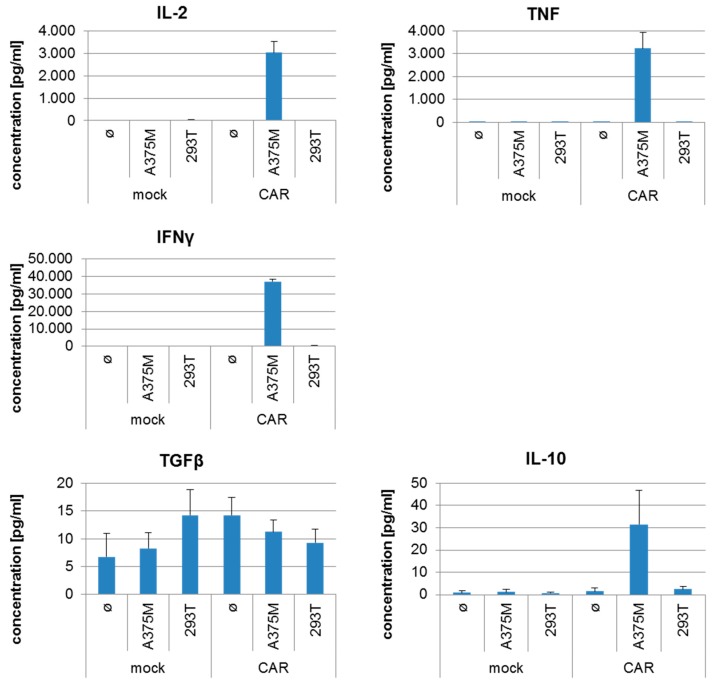
Antigen-specific cytokine production by mock- and CAR-transfected T cells. PBMCs were expanded, electroporated, and cryoconserved as described in Figure 1 in four consistency runs. As a control mock-electroporated cells were generated. After several days, the cells were thawed and used in a cytometric bead array. As target cells the CSPG4-negative 293T cell line and the CSPG4-positive A375M melanoma cell line were used. Target cells and effector cells were co-incubated for 20 h at a 1:1 ratio. As control, effector cells alone were also incubated (ø). Then supernatants were harvested and cytokine concentration was determined with a cytometric bead array. The mean cytokine secretions by transfected cells from the four consistency runs +/− SEM are shown. Please note the different scales.

Furthermore, we determined the antigen-specific cytokine production by the CAR-transfected T cells after incubation with the different target cells. As shown in Figure 7, the CSPG4-specific CAR-T cells recognized the CSPG4-positive melanoma cell line A375M and responded with the production of IL-2, TNF, and IFNγ, while the CSPG4-negative target cell line 293T was not recognized. Mock-transfected T cells did not recognize any of the target cell lines (Figure 7). Moreover, only background levels (~100 to 1000 fold less) of IL-10 and TGFβ, cytokines associated with regulatory T cells, were produced (Figure 7). 

In summary, these data show that our CSPG4-CAR-transfected T cells had a very high potency to lyse CSPG4-positive melanoma target cells, and produce cytokines antigen-specifically. We therefore conclude that these CAR-T cells produced at clinical scale can be used for the treatment of melanoma patients. 

The mean percentages of lysis by transfected cells from the four consistency runs +/− SEM are shown.

## 3. Discussion

CAR-T cells are a powerful tool in the fight against cancer. However, most published protocols on the generation of such cells are focusing on virally transduced CAR-T cells (reviewed in [56,57,58,59]). This study describes the establishment of the clinical-scale production of CAR-T cells for the treatment of melanoma patients by mRNA transfection of a CSPG4-specific CAR under full GMP in direct preparation of a clinical trial using these cells. Our production process was robust and resulted in a sufficient number of CSPG4-CAR-transfected T cells with a high potency to kill melanoma target cells.

This protocol allows the generation of 2.4 × 10^9^ CAR-expressing T cells starting from 1.77 × 10^8^ PBMCs. This is a considerable improvement in the yield of expanded and electroporated T cells in comparison to our previously published protocol in which only 6.7 × 10^8^ CAR-expressing T cells could be produced [54]. To limit the duration of the electroporation procedure, a maximum of 3.24 × 10^9^ cells were electroporated in 36 cuvettes, which was the maximum that could be practically handled. Increasing the number of cuvettes any further would lead to a too long duration of the procedure, with negative influences on mRNA integrity and cell viability. Notwithstanding, the protocol presented here can easily be scaled up even more, since we only used approximately 1/20 of PBMCs isolated from one leukapheresis, resulting in a theoretical possible number of 48 × 10^9^ CAR-expressing T cells. To be able to electroporate larger numbers of T cells with CAR-encoding mRNA, a semi-automated (flow-through) electroporation system should be used. Such systems are available from several companies, but their acquisition is very cost intensive. Additionally, the costs for the needed quantities of mRNA produced under full GMP compliance, using such a flow-through electroporation system, are relatively high compared to the production of a retroviral vector. Furthermore, compared to the previously published protocol [54], the handling was improved greatly and costs were reduced by: (1) usage of culturing bags instead of cell factories, (2) reduction of the duration of the production from ten to nine days, (3) reduction of manipulation by personnel during the production for one day (i.e., no handling on day three), and (4) reduction of the used quantity of IL-2 by changing the number of administrations from five to four times and reducing the IU/mL given at later time points. Therefore, our production protocol is suitable for the generation of a sufficient number of CAR-T cells with a standard electroporation device at considerably reduced expenses, which are affordable for many laboratories. The most important difference, however is that the previously published protocol [54] was a GMP-compliant protocol established and performed in a “normal” lab, while the protocol published here was indeed performed under full GMP in a licensed GMP-facility in full scale, meeting the corresponding QC-criteria. 

Some groups use both anti-CD3 and anti-CD28 antibodies for T-cell activation/expansion. However, in a previous publication [54], we showed that the addition of anti-CD28 antibody during the expansion of the T cells did not result in an improved expansion of these cells. Furthermore, the addition of the anti-CD28 antibody during the expansion of the T cells did not have any positive effect on the functionality of T cells after they were transfected with a CAR (i.e., they did not produce more cytokines or were more lytic after antigen-specific stimulation). Therefore, we have not included anti-CD28 antibody during expansion in the protocol presented here.

The remarkable clinical results with Kymriah^®^ (Tisagenlecleucel) and Yescarta^®^ (Axicabtagen-Ciloleucel) show the tremendous potency of CAR-T cells. However, depending on the target antigen, the potency of this approach can take a turn for the worse, as shown in a clinical trial with renal cell carcinoma patients. The chosen antigen, carbonic anhydrase IX, turned out to be expressed not only on the tumor cells, but also on the bile ducts. This resulted in an on-target/off-tumor reaction and grade two to four liver toxicity [60]. The fact that the transgenic receptor was introduced by retroviral transduction, leading to stable expression of this receptor, and constant reactivation of the transferred T cells encountering antigen, necessitated immunosuppressive treatment in these patients after cessation of T-cell transfer. As described in the introduction, it is very hard to find a target antigen for CARs on solid tumors that is not expressed on healthy tissue. Therefore, the approach of electroporation of receptor-encoding mRNA, that avoids all risks of retroviral transduction, was developed by the Rosenberg-group and our research group in parallel. Rosenberg and co-workers used TCR-RNA-transfected T cells for rapid screening of TCR functionality [61,62], and we were the first to show that TCR-RNA-transfected T cells were also able to lyse peptide loaded cells and, more importantly, tumor cell lines [63]. From our previous studies in which we efficiently transfected CD4^+^ and CD8^+^ T cells with CARs specific for ErbB2 (Her-2/neu) and carcinoembryonic antigen (CEA), we know that the CAR expression was transient with half-maximal expression at day two and no detectable CAR expression at day nine after electroporation [64]. The RNA-transfected T cells retained their cytolytic function after two days of activation and exhibited cytolytic activities similar to retrovirally transduced T cells. RNA electroporation of T cells thereby provides a versatile tool for transient CAR expression, which is of advantage in avoiding the persistence of unintended auto-reactivity. Consequently, for safety reasons we want to use CSPG4-specific CAR-T cells which are generated by mRNA electroporation in our clinical trial. As soon as we are convinced that on-target/off-tumor reactions are absent or controllable after careful monitoring of the patients, we can decide to stably introduce the CAR into the T cells. 

Such a strategy is also applied by others in clinical trials in patients with solid tumors using c-MET as a CAR-target antigen on breast cancer and melanoma [65], (NCT01837602; NCT03060356) and mesothelin as a CAR-target antigen on mesothelioma, pancreatic cancer, and ovarian cancer [66,67,68], (NCT03608618; NCT01897415; NCT01355965), or even with non-solid tumors using CD19 and CD123 as target antigen [69], (NCT02277522; NCT02624258; NCT02623582). The mRNA-CAR-T cells in these studies were well tolerated [65], the cells migrated to primary and metastatic tumor sites, showed a clinical anti-tumor activity, and showed no evidence of off-tumor on-target toxicity against normal tissues [66]. The clinical trials published by Beatty et al. and Maus et al., using mesothelin as antigen, showed a cytokine release syndrome in one mesothelioma patient resulting in adverse events (anaphylaxis, cardiac arrest, respiratory failure, disseminated intravenous coagulation) within minutes of completing the third infusion [66,67], while in pancreatic cancer patients no cytokine release syndrome and no dose-limiting toxicities, but actually stable disease in two patients were seen [68].

The disadvantage of using mRNA-transfected CAR-T cells is that the injection of the cells has to be repeated several times. Unlike retrovirally transduced cells, which have to be given only once and proliferate in the body of the patient, RNA-transfected cells will lose CAR expression and have to be replenished from the outside to maintain high cytolytic pressure on the tumor. The possible reason for the severe adverse events in the patient described above [66,67] was that the CAR was based on a murine antibody and the adverse event was caused by IgE antibodies specific to the CAR. These antibodies were probably induced by the intermittent dosing schedule of the CAR-T cells [66,67]. From this study and the dosing schedule of the latter Beatty study (three times weekly (M-W-F), for three weeks, intravenously), it seems best that infusions of CAR-T cells are not separated by more than 10 days, and are completed within 21 days.

The doses used in the clinical trials described above are varying greatly from between a single injection of 3 × 10^7^ cells to several injections of 5.25 × 10^8^ cells. Therefore, the total number of cells per patient used in these trials varies also between 3 × 10^7^ and 4.725 × 10^9^ cells. Considering that our clinical trial will use CSPG4-specific CAR-T cells for the first time in humans, we will perform a dose-escalation, and therefore we have cryoconserved our cells in relatively small batches of 45 × 10^6^ cells. We are convinced that 2.4 × 10^9^ cells are sufficient for the treatment of melanoma patients even when a three times weekly, three weeks schedule (i.e., a maximum of 2.66 × 10^8^ cells per injection for patients treated with the highest dose) is applied. 

Taken together, we believe that mRNA-based transfection of CARs, especially in the treatment of solid tumors, will emerge as a standard transfer method to ensure safety and to screen for adverse effects. The protocol for the generation of CAR-mRNA-transfected T cells described here is highly feasible, highly reproducible, and performed at a clinical-scale under full GMP compliance, and will be used for a clinical trial with CSPG4-specific CAR-T cells in melanoma patients.

## 4. Materials and Methods

### 4.1. Cells and Reagents

Peripheral blood mononuclear cells (PBMCs) were extracted from leukaphereses, obtained from healthy donors following informed consent and approved by the institutional review board (reference number: 251_16 B), using density centrifugation on Lymphoprep^TM^ (Axis-Shield, Oslo, Norway).

Target cell lines included the CSPG4-negative 293T cell line and the CSPG4-positive melanoma cell line A375M (kind gift from Dr. Aarnoudse, Leiden, The Netherlands). Target cells were cultured in R10 medium consisting of RPMI 1640 (Lonza, Basel, Switzerland) supplemented with 2 mM L-glutamine (Lonza), 100 IU/mL penicillin (Lonza), 100 mg/mL streptomycin (Lonza), 10% (v/v) heat-inactivated fetal calf serum (PAA, GE healthcare, Piscataway, NY, USA), 2 mM HEPES (PAA, GE healthcare), and 2 mM β-mercaptoethanol (Gibco, Thermo Fisher, Carlsbad, CA, USA).

### 4.2. T-Cell Expansion

A flow chart and a detailed description of the T-cell expansion procedure are provided in Supplementary Figure S2. In short: PBMCs were isolated from leukaphereses by density centrifugation using Lymphoprep^TM^ (Abbott-Diagnostics, Oslo, Norway), and were expanded for nine days in X-vivo 15 medium (GMP product, Lonza, Verviers, Belgium) in EVA bags (Macopharma, Langen, Germany) with a starting concentration of 1 × 10^6^ cells/mL. OKT-3 (0.1 µg/mL) (GMP product, Miltenyi Biotec, Bergisch-Gladbach, Germany) was added at day zero. IL-2 (Proleukin; Novartis, Nuremberg, Germany) was added at day zero (1000 IU/mL), day two (1000 IU/mL), day five (500 IU/mL), and day seven (250 IU/mL). Cells were diluted at day two to 2 × 10^5^ cells/mL, and at day five and seven the volume of the X-vivo 15 medium was doubled. At day nine, the cells were harvested and 3.24 ×10^9^ cells were electroporated with mRNA encoding the CSPG4-specific CAR (see Section 4.4). Four hours after that, the CAR-transfected T cells were cryoconserved in batches of 45 × 10^6^ cells (see Section 4.5).

### 4.3. In Vitro Transcription of mRNA

A flow chart and a detailed description of the in vitro transcription of mRNA are provided in Supplementary Figure S3. A second generation CAR (CSPG4_HL_-CD28/CD3ζ-CAR) directed against CSPG4 (chondroitin sulfate proteoglycan 4) was used for transfer into T cells. The molecular composition of the receptor was specified previously [52]. The CSPG4-CAR-encoding RNA is manufactured from a template-plasmid containing the complementary sequence. The plasmid contains a T7-promotor sequence that is utilized in the IVT-process. The template plasmid is obtained in a circular format. Linearization is required to avoid infinite transcription round the circle. Hence, the plasmid is digested with the restriction-endonuclease *Spe*I. The linearized plasmid is purified using chloroform and isoamyl-alcohol, then precipitated with potassium acetate and ethanol, and dried and dissolved in nuclease-free water. The linearized plasmid is tested for integrity, yield, concentration, and purity. In vitro transcription of receptor-encoding mRNA was executed with T7 RNA polymerase (mMESSAGE mMACHINE T7 Ultra kit; Thermo Fisher, Carlsbad, CA, USA) according to the manufacturer’s instructions. Afterwards, mRNA was purified on RNeasy columns (Qiagen GmbH, Hilden, Germany) according to the manufacturer’s instructions. Before use, the mRNA quality was assessed by agarose gel electrophoresis.

### 4.4. RNA Electroporation

A flow chart and a detailed description of the electroporation of expanded T cells with mRNA encoding the CSPG4-specific CAR are provided in Supplementary Figure S4. RNA transfection was performed as detailed elsewhere [63]. In short, following expansion, T cells were washed in OptiMem (Thermo Fisher, Carlsbad, CA, USA,) resuspended at 150 × 10^6^ cells/mL OptiMem and transferred to 4-mm gap electroporation cuvettes (Biolab Products, Bebensee, Germany). Cells were either mock-electroporated or transfected with 150 μg/mL of RNA coding for the CSPG4-specific CAR (CSPG4_HL_-CD28-CD3ζ) using a Gene Pulser Xcell (Bio-Rad, Hercules, CA, USA) at 500 V (square wave pulse) for 5 ms. After transfection, T cells were cultured 3–4 h in X-vivo 15 medium at a concentration of 2 × 10^6^ cells/mL.

### 4.5. Cryoconservation of Electroporated T Cells

CAR-transfected T cells were cryoconserved in batches of 45 × 10^6^ cells per vial at a final concentration of 25 × 10^6^/mL in Human Serum Albumin solution (Baxter, Heidelberg, Germany) containing 5% Glucose (Glucosteril, B.Braun, Melsungen, Germany) and 10% DMSO (Sigma Aldrich, St.Louis, MO, USA).

### 4.6. Flow Cytometry

Cellular composition during and after expansion was analyzed on day zero, two, seven, and nine with anti-CD3 (BD Biosciences, San Jose, CA, USA, CE/IVD, clone: SK7) and anti-CD8 (BD Biosciences, USA, CE/IVD, clone: SK1), anti-CD25 (BD Biosciences, USA, clone: M-A251), anti-CD19 (BD Biosciences, USA, CE/IVD, clone: SJ25C1), anti-CD56 (BD Biosciences, USA, CE/IVD, clone: NCAM16.2) anti-CD16 (BD Biosciences, USA, clone: 3G8) antibodies. Isotype-stained cells served as controls. The same antibodies were used to determine cell phenotype after expansion, electroporation, and cryoconservation. 

Surface expression of the introduced CSPG4-specific CAR was analyzed flow cytometrically after thawing of cryoconserved cells. The CAR was stained with goat-F(ab’)2 anti-human IgG antibody (Southern Biotech, Birmingham, AL, USA) directed against the extracellular IgG1 CH2CH3 (Fc-spacer) CAR-domain. 

Immunofluorescence was measured using a FACSCalibur cytofluorometer (BD Biosciences, Heidelberg, Germany) equipped with the CellQuest software (BD Biosciences). Data were analyzed using the FCS Express 5 (De Novo Software, Glendale, CA, USA).

### 4.7. Cytotoxicity Assay

For cytotoxicity assays, the CSPG4-negative 293T cell line and the CSPG4-positive A375M melanoma cell line were used as target cells. 293T cells were labeled with 0.25 µM CFSE (Thermo Fisher, Carlsbad, CA, USA) and A375M cells were labeled with 2.5 µM CFSE according to Noto et al. [70]. These cells were mixed at a 1:1 ratio. Target cells and effector cells were co-incubated for 20 h at indicated target: effector ratios at 1 × 10^6^ of total cells in 1 mL of X-Vivo 15 medium containing 2% human serum (Sigma Aldrich, St. Louis, MO, USA) in a 48-well plate (BD Biosciences, USA) in triplicates. Then, cells were harvested and stained with 7-AAD (BD Biosciences, USA). CFSE and 7-AAD staining was determined by flow cytometry. The percentages of living A375M cells (i.e., 7-AAD negative and CFSE^high^) were determined. The percentage of lysis (i.e., cytotoxicity induced by T cells and background apoptosis) of A375M cells was calculated as follows, with A being:

$$A\;=\frac{\%\;living\;A375M\;cells}{\%\;living\;293T\;cells}$$

and percentage of lysis being:

$$\frac{A\;\%\;\left[target\;alone\right]-A\;\%\;\left[target\;+\;effector\right]}{A\;\%\;\left[target\;alone\right]}\begin{array}{l} \times \;100\% \end{array}$$

### 4.8. Cytokine Secretion

Cytokine secretion by CSPG4-CAR T cells in response to target cells was assayed with a cytometric bead array. Mock-electroporated T cells served as control. T cells were stimulated over-night at a 1:1 ratio with 293T and A375M cells. The supernatants were recovered and the concentrations of the indicated cytokines were quantified using the Th1/Th2 Cytometric Bead Array Kit II and Human TGFβ CBA Flex Set (both BD Biosciences) in accordance with the manufacturer’s instructions. Immunofluorescence was measured with the FACSCanto (BD Biosciences, Franklin Lakes, NJ, USA) operating with FACSDiva software (BD Biosciences). Data analysis was carried out using the FCS Express 5.

## 5. Conclusions

In this study we describe the establishment of the clinical-scale production of CAR-T cells for the treatment of melanoma patients by mRNA transfection of a CSPG4-specific CAR under full GMP compliance in direct preparation of a clinical trial using these cells. Four consistency runs using the optimized protocol for CAR-T cell production proofed the stable production of a sufficient number of CSPG4-CAR-transfected T cells with a low contamination by other cells, a very high transfection efficiency, a high CAR expression, and a high potency to kill melanoma target cells.

## Figures and Tables

**Table 1:** Results of expansion and electroporation of T cells originating from PBMCs ^1^ isolated from leukaphereses.

Run	^2^ PBMCs Day 0	Cell Number Harvested at Day 9	Maximum Number of Cells Electro-Porated at Day 9	CAR-Transfected T Cells at Day 9	Survival after Electro-Poration	Survival after Electroporation and Cryo-Conservation	Yield Factor
Con 1	292	4876	3240	2278	70.3%	55.1%	7.8
Con 2	231	3996	3240	2456	75.8%	61.4%	10.6
Con 3	85	5466	3240	2450	75.6%	61.4%	28.8
Con 4	341	4344	3240	2424	74.8%	55.6%	7.1
Average	237.3	4670.5	3240	2402	74.1%	58.4%	13.6
SD	111.0	642.0		83.8	2.6%	3.5%	10.3

^1^ peripheral blood mononuclear cells; ^2^ all cell numbers ×10^6^.

## Supplementary Materials

The following are available online at https://www.mdpi.com/2072-6694/11/8/1198/s1, Figure S1: Viability of CSPG4-CAR-RNA-transfected T cells after cryoconservation. Figure S2: Preferential expansion of T cells from PBMCs isolated from leukapheresis products. Figure S3: In vitro transcription of template DNA and purification of IVT-RNA. Figure S4: Electroporation of expanded T cells with mRNA encoding a CSPG4-specific CAR. Figure S5: CD4-positive fraction of the CAR-transfected cells after expansion over 9 days, electroporation and cryoconservation. Figure S6: Gating strategy of the cytotoxicity assay. Table S1: Original data used for Figure 2. Table S2: Original data used for Figure 3. Table S3: Original data used for Figure 4a. Table S4: Original data used for Figure 6. Table S5: Original data used for Figure 7. Table S6: Original data used for Supplemental Figure S1. Table S7: Original data used for supplemental figure S5.
