# Supplementary material for: Clinical-Scale Production of CAR-T Cells for the Treatment of Melanoma Patients by mRNA Transfection of a CSPG4-Specific CAR under Full GMP Compliance
Source: Cancers (Basel). 2019 Aug 16;11(8):1198. doi: 10.3390/cancers11081198 (PMC6721485; doi:10.3390/cancers11081198)
Supplement: Supplementary file 1 [file cancers-11-01198-s001.pdf]

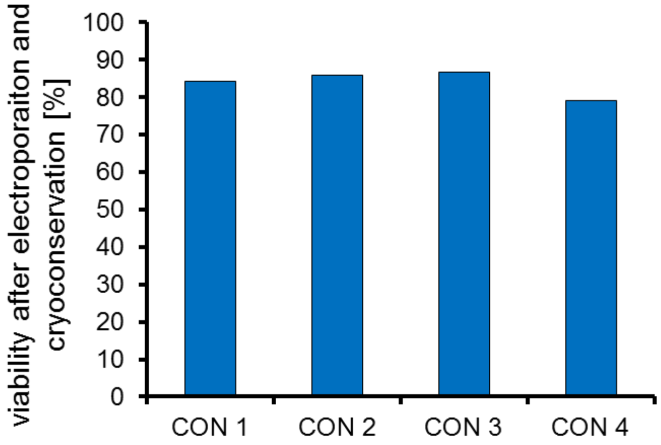

Supplementary figure S1: Viability of CSPG4-CAR-RNA-transfected T cells after cryoconservation. PBMCs were expanded, electroporated, and cryoconserved as described in figure 1 in four consistency runs. After several days, the cells were thawed and cell viability was determined by trypan blue staining. The percentages of viable cells of all 4 consistency runs are shown.

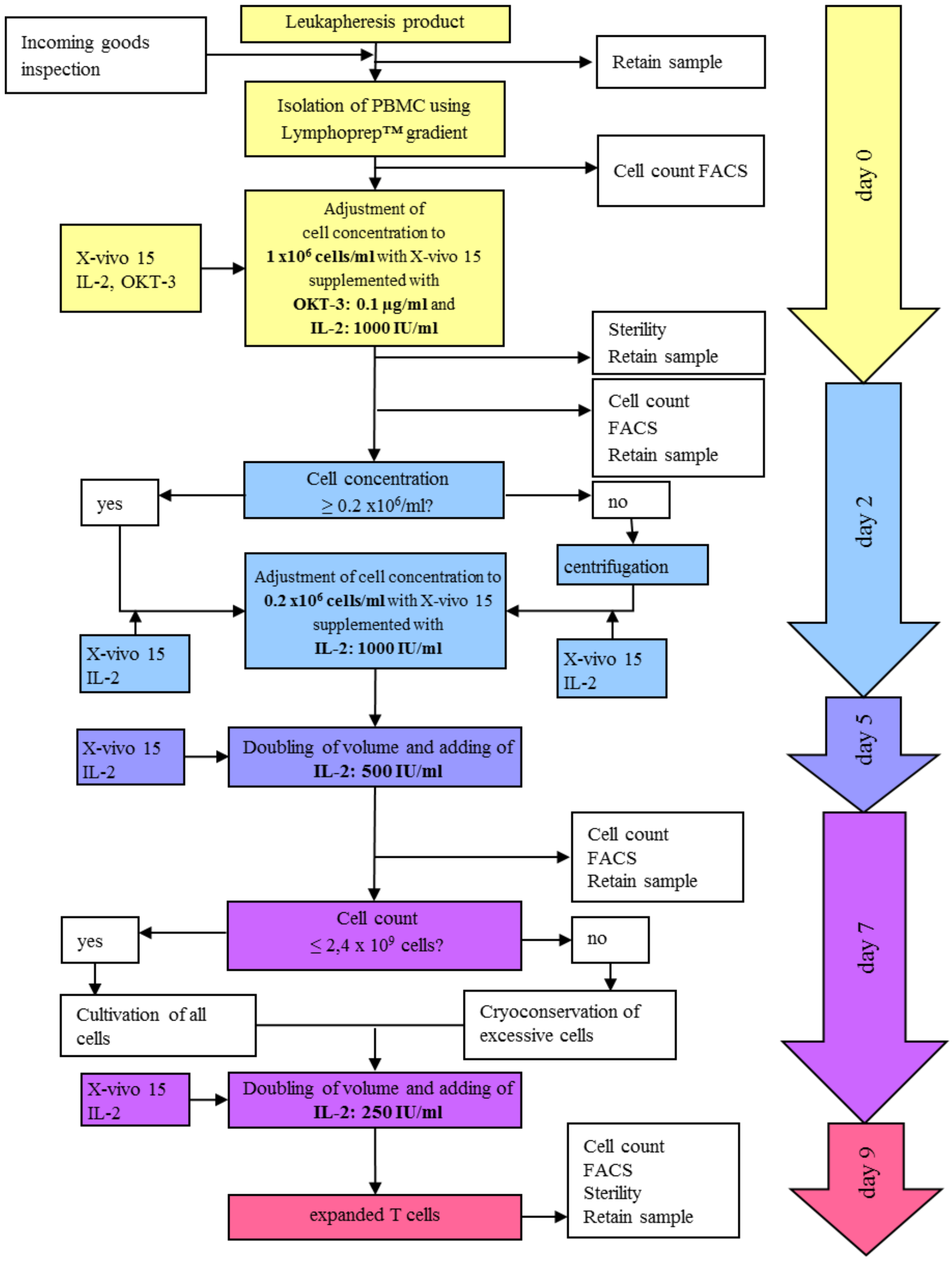

Supplementary figure S2: Preferential expansion of T cells from PBMCs isolated from leukapheresis products.

Leukaphereses products are obtained from the blood bank and are acquired from healthy donors following informed consent. From the leukapheresis a sample is retained.

On day 0, PBMCs are isolated from leukaphereses by density centrifugation using LymphoprepTM. In short: the leukapheresis is diluted with Dulbecco's Phosphate Buffered Saline (PBS) with 10% Acid-Citrate-Dextrose (ACD). This suspension is layered on top of Lymphoprep™, being careful to minimize mixing of blood with Lymphoprep™. The tubes are centrifuged at 500g for 30 minutes at room temperature. The upper plasma layer is removed and discarded without disturbing the plasma:Lymphoprep™ interface. The PBMC layer is removed at the plasma:Lymphoprep™ interface without disturbing the erythrocyte/ granulocyte pellet, and washed several times with PBS with Ethylenediaminetetraacetic acid (EDTA). Cells are counted by trypan blue staining and a phenotype determination is done by flow cytometry (Ph. Eur. 2.7.24), measuring CD3- (T cells), CD8- (CD8+ T cells), CD16/CD56- (NK cells), CD19- (B cells), CD14- (monocytes), and CD25-expression for information only. Cells are then diluted to a concentration of $1x10^6$ cells/ml in X-vivo 15 (a chemically defined, serum-free, hematopoietic cell medium, with L-glutamine, and without gentamicin or phenol red), supplemented with 0.1 µg/ml OKT-3 and 1000 IU/ml IL-2. PBMCs obtained by density gradient centrifugation

are cultivated *in vitro* in bags. Cell cultures are gently mixed and incubated (37 °C ± 1 °C, 5 % ± 1 % CO_2, > 70 % r.h.). A sample for sterility testing according to Ph. Eur. 2.6.27 is taken from the isolated PBMCs.

On day 2, cell count is determined by trypan blue staining. The cell concentration is adjusted to 0.2×10^6/ml with fresh X-vivo 15 medium. In order to substitute cellular consumption, fresh IL-2 (1000 IU/ml) is added. Cell cultures are gently mixed and incubated (37 °C ± 1 °C, 5 % ± 1 % CO_2, >70 % r.h.). The composition analysis is done by flow cytometry, measuring again CD3-, CD8-, CD16/CD56-, CD19-, CD14-, and CD25-expression for information only.

On day 5, the volume of culture medium is doubled by adding fresh X-vivo 15 medium. Additionally, fresh IL-2 (500 IU/ml) is added. Cell cultures are gently mixed and incubated (37 °C ± 1 °C, 5 % ± 1 % CO_2, >70 % r.h.).

On day 7, cell count is determined by trypan blue staining. If 2.4×10^9 or less cells are present in the culture, all cells are taken for further culturing. If more than 2.4×10^9 cells are present in the culture, only 2.4×10^9 cells are continued in culture and the remainder of the cells is frozen. The composition analysis is done by flow cytometry, measuring again CD3-, CD8-, CD16/CD56-, CD19-, CD14-, and CD25-expression for information only. The volume of culture medium is doubled by adding fresh X-vivo 15 medium. Additionally, fresh IL-2 (250 IU/ml) is added. Cell cultures are gently mixed and incubated (37 °C ± 1 °C, 5 % ± 1 % CO_2, >70 % r.h.).

On day 9, the expanded T cells are collected, centrifuged, resuspended in fresh X-Vivo 15 and evaluated for cell number and cell viability by trypan blue staining (Ph. Eur. 2.7.29), as well as cell phenotype, identity and cell purity by flow cytometry (Ph. Eur. 2.7.24; measuring CD3-, CD8-, CD16/CD56-, CD19-, CD14-, and CD25-expression) before electroporation with CSPG4-CAR-mRNA (see supplemental figure S4). Furthermore, sterility of the expanded T cells is tested. If 3.24×10^9 or less cells are present in the culture, all cells are taken for further processing (i.e. electroporation). If more than 3.24×10^9 cells are present in the culture, only 3.24×10^9 cells are electroporated and the remainder of the cells is frozen.

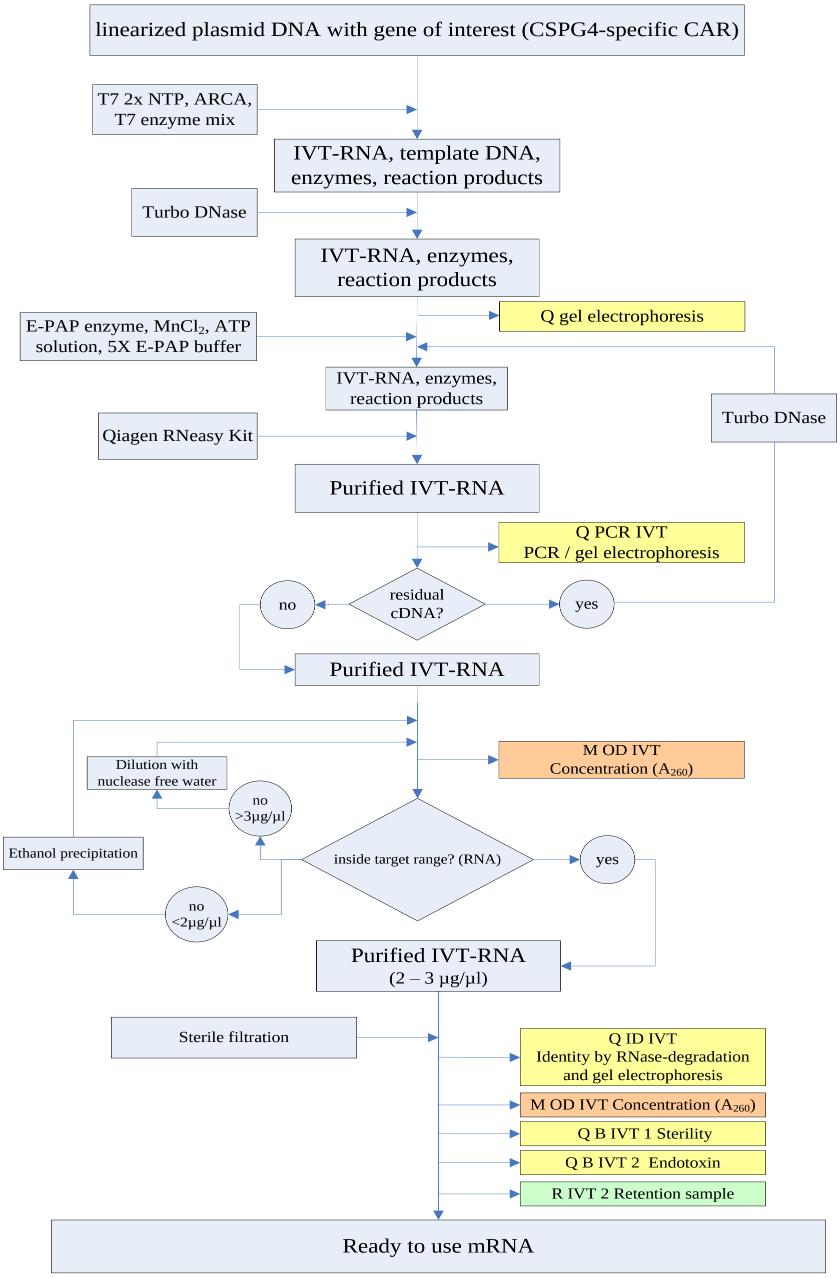

Supplementary figure S3: *In-vitro* transcription of template DNA and purification of IVT-RNA.
The CSPG4-CAR-RNA is enzymatically *in vitro* transcribed from the linearized plasmid template using RNA-polymerase from bacteriophage T7, which is suitable for the binding on the introduced T7 promotor sequence of the plasmid DNA. In combination with the cap analog, anti-reverse cap analog (ARCA), transcribed mRNA is capped for generating functional and translatable *in vitro* transcribed CSPG4-CAR messenger RNA (CSPG4-CAR-IVT-RNA), which is further purified. For *in vitro* transcription the following reagents are added: T7 2xNTP, ARCA cap, T7 enzyme mix. The solution is mixed and incubated at 37 °C ± 2 °C for 3 h to 3 h 15 min. Turbo DNase is added to remove the DNA template. The solution is mixed and incubated for 2 hours at 37° C. Then E-PAP enzyme, $MnCl_2$, ATP solution, and 5x E-PAP buffer are added for the polyadenylation of the *in vitro* transcribed RNA. A sample on non-polyadenylated IVT-RNA is stored for gel electrophoresis. The resulting IVT-RNA is purified using the RNeasy maxi Kit of Qiagen. The purified IVT-RNA is tested for residual cDNA by PCR and gel electrophoresis. If residual cDNA is present, the DNase digestion is repeated. By OD measurement, the concentration of the purified IVT-RNA is determined. Dilution with nuclease free water or ethanol precipitation is performed to obtain an IVT-RNA concentration of 2-3 µg/µl. Then sterile filtration is performed on the IVT-RNA. Quality control samples for identity (RNase-degradation), sterility,

and endotoxin contamination are stored. Furthermore, the concentration of the filtered IVT-RNA is determined, and a retention sample is stored.

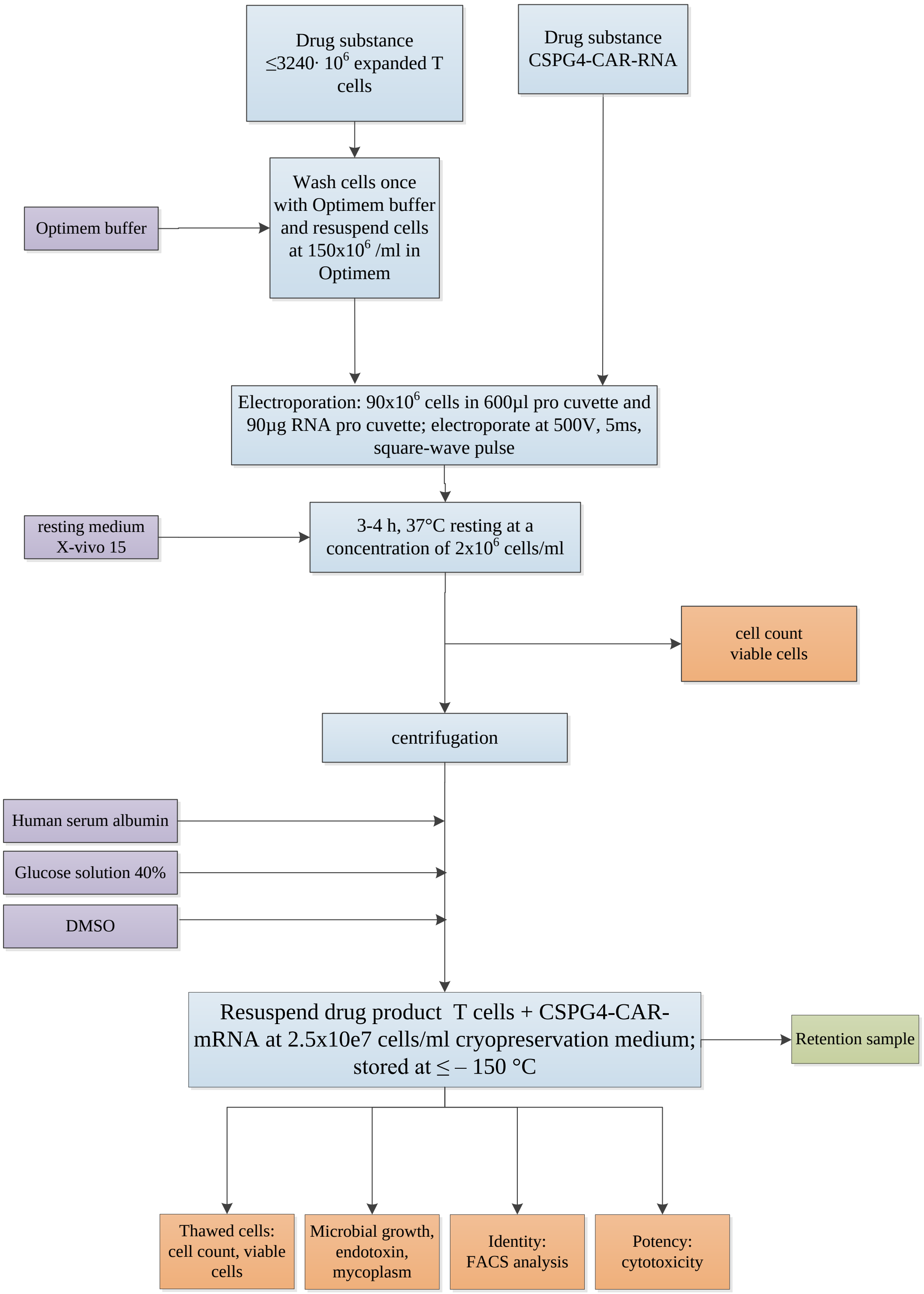

Supplementary figure S4: Electroporation of expanded T cells with mRNA encoding a CSPG4-specific CAR.

Prior to electroporation, a resting medium is freshly prepared in a bag. The total volume is calculated on the basis of used cuvettes for electroporation (45 ml resting medium per cuvette containing $90x10^6$ viable cells for electroporation). The resting medium is pure X-vivo 15 medium. The expanded T cells are washed once in minimal essential medium, OPTI-MEM®. Viable cell concentration is adjusted to a concentration of $90x10^6$ viable cells per 600 µl volume (i.e. $150x10^6$ cells/ml). Electroporation is done using special cuvettes and

adequate instrumental settings of the Gene Pulser (Xcell, BioRad). The number of cuvettes is calculated based on cell count. Per cuvette $90x10^6$ viable cells are used, and a maximum of 3240 $x10^6$ cells are electroporated. Cuvettes are loaded with 90 µg CSPG4-CAR-IVT-RNA. To each cuvette 600 µl cell suspension is added and the cuvette is placed into the Gene Pulser. The suspension is electroporated with one pulse at 500 V for 5 ms using a square-wave pulse. Directly after electroporation, the suspension is transferred into resting medium. The cuvette is washed twice with resting medium. This procedure is repeated according to the number of cuvettes. Electroporated cells are incubated for 3 h to 4 h in an incubator (37 °C ± 1 °C, 5 % ± 1 % CO2, >70 % r.h.).

Then electroporated cells are harvested and re-suspended in X-vivo 15 medium. Cell count and viability are determined, cells are centrifuged, and re-suspended at 2°C to 8°C in the appropriate volume of HSA to a concentration of $50x10^6$ viable cells per ml. 900 µl cell suspension are filled into each cryovial at 2°C to 8°C. After adding 900 µl freezing medium (55.0 Vol.-% HSA, 20 Vol.-% DMSO, 10 % glucose) to every vial, the closed vials are mixed gently and transferred immediately into a freeze container. The freeze container is than stored immediately at -75 °C ± 10 °C for 4 to 18 h. Subsequently, the vials are transferred to the gas phase of liquid nitrogen (≤ -150 °C).

Thawed cells are counted and viability is determined. Furthermore, microbial growth, and endotoxin and mycoplasm contamination are determined. CSPG4-CAR expression is investigated by flow cytometry. The potency of the CSPG4-CAR-transfected T cells is tested in a cytotoxicity assay.

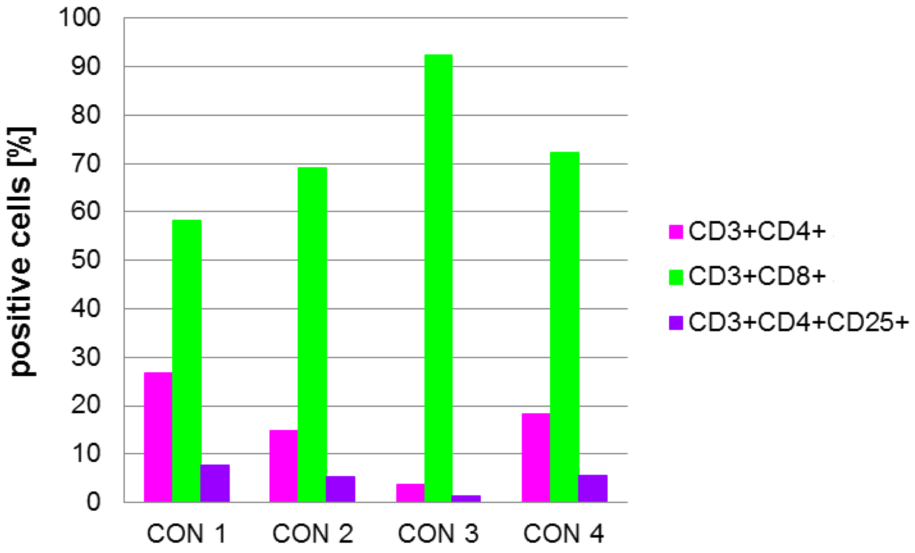

Supplementary figure S5: CD4-positive fraction of the CAR-transfected cells after expansion over 9 days, electroporation and cryoconservation.

PBMCs were expanded, electroporated, and cryoconserved as described in figure 1 in four consistency runs. After several days, the cells were thawed, and 4 hours after thawing the phenotype of the cells was determined by measuring CD3, CD4, CD8, and CD25 expression using corresponding antibodies. The percentages of positive cells in the live gate of all 4 consistency runs are shown.

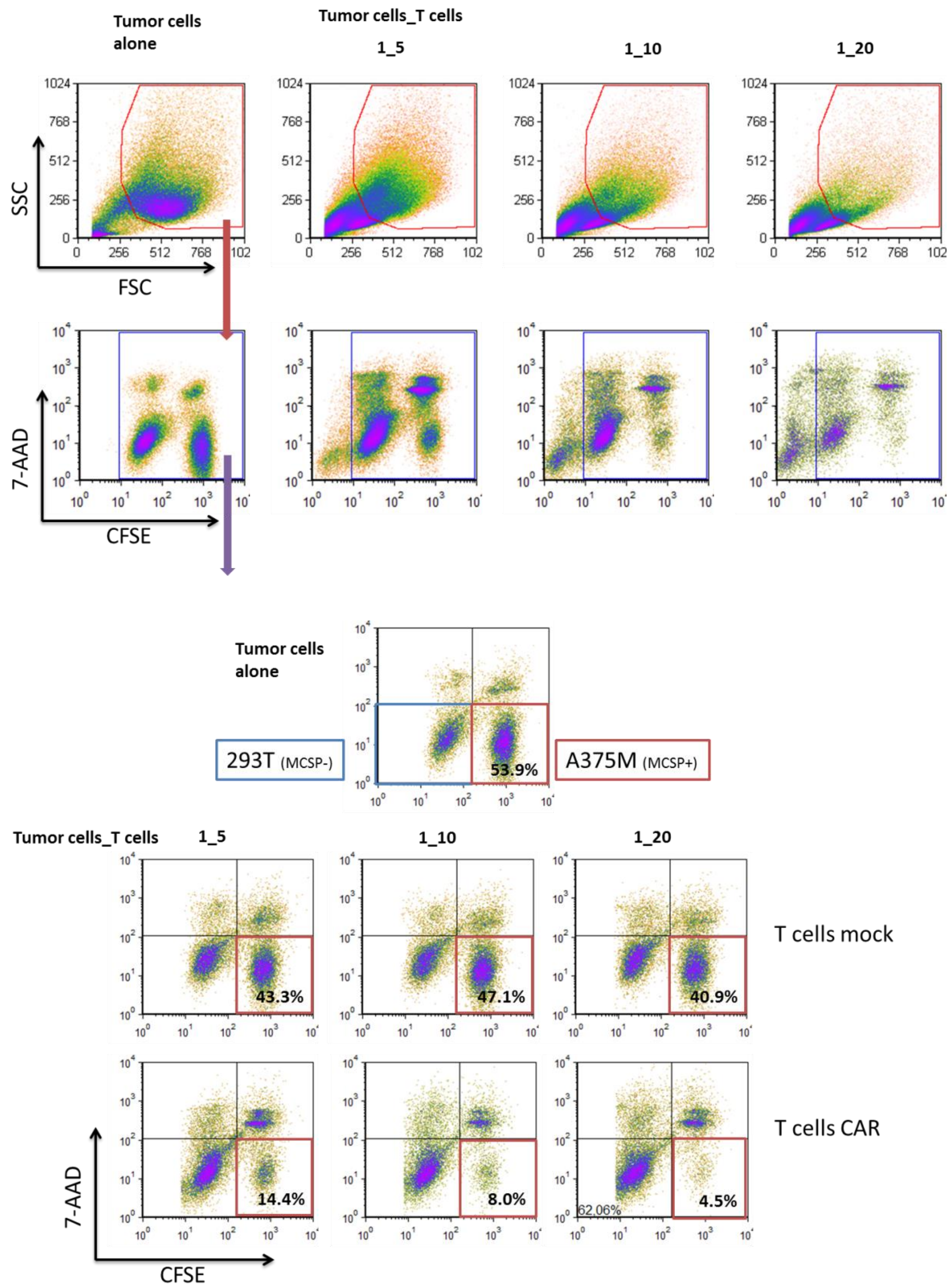

Supplementary figure S6: Gating strategy of the cytotoxicity assay.

For cytotoxicity assays the following gating strategy was performed: first tumor cells were gated in forward and side scatter. As the tumor cells are larger than the T cells, this already excludes most of the latter. Then in the 7-AAD, CFSE measurement a gate on CFSE positive cells (i.e. the two tumor cell populations, but not the T cell population, which is CFSE negative) was made, resulting in the depicted data.

Raw data used for this manuscript

Supplementary table S1: Original data used for Figure 2

		CD3+[1]	CD3+CD8+	CD3+CD25+	CD3-CD19+	CD3-CD56/16+
**Day 0**	CON1	20.3	3.9	0.7	12.8	12.8
	CON2	49.5	11.4	1.3	10.2	9.3
	CON3	43.7	23.9	0.2	6.0	19.9
	CON4	29.2	6.4	13.3	10.9	19.4
**Day 2**	CON1	38.2	12.5	32.3	10.0	37.5
	CON2	77.1	23.9	76.0	12.4	7.7
	CON3	74.7	40.1	60.2	6.0	17.7
	CON4	36.5	11.1	30.2	12.7	39.7
**Day 7**	CON1	93.1	58.1	69.3	1.3	3.7
	CON2	96.3	64.3	42.4	0.6	0.6
	CON3	98.3	93.0	21.6	0.1	0.5
	CON4	93.6	68.7	65.9	1.1	3.4
**Day 9**	CON1	97.2	66.5	33.1	0.3	1.9
	CON2	98.0	68.4	32.7	0.7	0.1
	CON3	99.3	91.4	14.1	0.1	0.6
	CON4	96.2	73.6	21.3	0.1	2.7
**Average**	**day 0**	35.7	11.4	3.9	10.0	15.4
	**day 2**	56.6	21.9	49.7	10.3	25.7
	**day 7**	95.3	71.0	49.8	0.8	2.1
	**day 9**	97.7	75.0	25.3	0.3	1.3
**SEM[2]**	**day 0**	6.7	4.4	3.1	1.4	2.6
	**day 2**	11.1	6.7	11.1	1.5	7.8
	**day 7**	1.2	7.6	11.1	0.3	0.9
	**day 9**	0.7	5.7	4.6	0.1	0.6

[1] Indicated are percentages positive cells.

[2] SEM=standard error of the mean.

Supplementary table S2: Original data used for figure 3

		CD3+[1]	CD3+CD8+	CD3+CD25+	CD3-CD19+	CD3-CD56/16+
**Run**	CON1	98.4	68.1	39.5	0.1	1.7
	CON2	98.7	75.1	50.4	0.5	0.8
	CON3	99.6	93.5	9.0	0.0	0.5
	CON4	98.0	75.6	19.9	1.8	1.8
**Average**		98.7	78.1	29.7	0.6	1.2

[1] Indicated are percentages positive cells.

Supplementary table S3: Original data used for figure 4a

		CAR expression[1]
**Run**	CON1	93.6
	CON2	81.9
	CON3	85.5
	CON4	81.5
**Average**		85.6

[1] Indicated are percentages positive cells.

Supplementary table S4: Original data used for figure 6

	**tumor : T cell ratio**	**1:2**	**1:3**	**1:5**	**1:10**	**1:20**
**CON1**	CAR-transf.	76.9	89.0	92.2	93.6	94.6
**CON2**	CAR-transf.	59.2	76.5	85.8	91.5	92.3
**CON3**	CAR-transf.	45.2	68.9	80.3	87.0	89.1
**CON4**	CAR-transf.	32.1	49.9	68.5	87.8	93.2
**CON1**	mock-transf.	20.9	22.7	47.7	52.0	52.3
**CON2**	mock-transf.	-6.4	-10.9	8.5	12.1	24.5
**CON3**	mock-transf.	5.9	-7.1	-23.5	3.6	17.3
**CON4**	mock-transf.	-78.7	-64.1	-59.8	-39.9	-9.3
**Average**	CAR-transf.	53.4	71.1	81.7	90.0	92.3
**Average**	mock-transf.	-14.6	-14.9	-6.8	7.0	21.2
**SEM[2]**	CAR-transf.	9.6	8.2	5.0	1.5	1.2
**SEM**	mock-transf.	22.1	18.1	22.9	18.9	12.7

[1] Indicated are percentages of lysis.

[2] SEM=standard error of the mean.

Supplementary table S5: Original data used for figure 7

**Probe**	**IL-2 pg/ml**	**IL-10 pg/ml**	**TNF pg/ml**	**IFNγ pg/ml**	**TGFß pg/ml**
C1-mock alone	3	0	1	11	1
C1-mock A375M	6	4	6	207	6
C1-mock 293T	26	1	10	143	4
C1-MCSP alone	2	0	2	9	9
C1-MCSP A375M	4483	77	4247	40346	11
C1-MCSP 293T	10	6	7	249	11
C2-mock alone	1	0	1	6	8
C2-mock A375M	16	0	9	141	2
C2-mock 293T	35	0	4	311	25
C2-MCSP alone	1	0	2	14	10
C2-MCSP A375M	2347	17	2500	36525	17
C2-MCSP 293T	37	0	43	835	2
C3-mock alone	3	0	3	15	0
C3-mock A375M	14	0	6	235	10
C3-mock 293T	20	0	5	79	10

C3-MCSP alone		12	0	3	14	23
C3-MCSP A375M		2994	17	4529	37620	10
C3-MCSP 293T		19	0	12	89	12
C4-mock alone		2	3	4	27	18
C4-mock A375M		13	0	12	104	15
C4-mock 293T		32	1	38	527	18
C4-MCSP alone		4	6	2	27	15
C4-MCSP A375M		2284	15	1686	33884	7
C4-MCSP 293T		6	3	6	74	12
Average		IL-2 pg/ml	IL-10 pg/ml	TNF pg/ml	IFNγ pg/ml	TGFß pg/ml
mock	ø	2	1	3	15	7
	A375M	12	1	8	171	8
	293T	28	1	14	265	14
CAR	ø	5	2	2	16	14
	A375M	3.027	32	3.241	37.094	11
	293T	18	2	17	312	9
SEM						
mock	ø	0,4	0,8	0,7	4,5	4,2
	A375M	2,3	1,1	1,5	29,9	2,8
	293T	3,4	0,3	7,9	100,2	4,6
CAR	ø	2,6	1,6	0,4	3,8	3,2
	A375M	511,0	15,3	685,6	1338,0	2,1
	293T	6,8	1,3	8,7	178,9	2,4

Supplementary table S6: Original data used for supplemental figure S1

		**Surviving cells of 45x10^6 frozen cells**	**Viability**
**Run**	CON1	37.9[1]	84.2[2]
	CON2	38.7	86.0
	CON3	39.0	86.7
	CON4	35.6	79.1
**Average**		37.8	84.0

[1] Indicated cell number x10^6.

[2] Indicated are percentages of cells.

Supplementary table S7: Original data used for supplemental figure S5

	**CD3+CD4+**	**CD3+CD8+**	**CD3+CD4+CD25+**
**CON 1**	26.9[1]	58.2	7.6
**CON 2**	15.0	69.2	5.4
**CON 3**	3.6	92.5	1.4
**CON 4**	18.3	72.3	5.6
**Average**	15.9	73.1	5.0

[1] Indicated are percentages of cells.
